# The *PIN2* ortholog in barley modifies root gravitropism and architecture

**DOI:** 10.1002/tpg2.70061

**Published:** 2025-08-06

**Authors:** Zachary Aldiss, Yasmine Lam, Hannah Robinson, Richard Dixon, Laura Steinhardt, Peter Crisp, Ian Godwin, Andrew Borrell, Lee Hickey, Karen Massel

**Affiliations:** ^1^ Queensland Alliance for Agriculture and Food Innovation The University of Queensland Brisbane Queensland Australia; ^2^ InterGrain Pty Ltd Perth Western Australia Australia; ^3^ School of Agriculture and Food Sustainability The University of Queensland Brisbane Queensland Australia; ^4^ Queensland Alliance for Agriculture and Food Innovation The University of Queensland Warwick Queensland Australia

## Abstract

Roots provide the critical interface where plants acquire nutrients and water, but our limited understanding of the genetic controls modulating root system architecture (RSA) in crop species constrains opportunities to develop future cultivars with improved root systems. However, there is vast knowledge of root developmental genes in model plant species, which has the potential to accelerate progress in crops with more complex genomes, particularly given that genome editing protocols are now available for most species. *PIN‐FORMED2* (*PIN2*) encodes a root‐specific polar auxin transporter, where its absence resulted in roots being unable to orient themselves using gravity, producing a significantly wider root system. To explore the role of *PIN2* in a cereal crop, we used CRISPR/Cas9 (where CRISPR is clustered regularly interspaced short palindromic repeats) editing to knockout of *PIN2* in barley (*Hordeum vulgare*). Like Arabidopsis, the roots of barley *pin2* loss‐of‐function mutants displayed an agravitropic response at seedling growth stages, resulting in a significantly shallower and wider root system at later growth stages. Notably, despite the significant change in RSA, there was no change in shoot architecture or total shoot biomass, with an insensitivity to the effects of higher planting density. We discuss the future challenges and opportunities to harness the *PIN2* pathway to optimize RSA in crops for a range of production scenarios without a shoot trade‐off.

## Core Ideas

- *PIN‐FORMED* (PIN2) regulates root system architecture in barley.
- Gene editing was used to create novel genetic variants.
- Outcomes show wider root systems without impacting canopy development.
- In‐depth root phenotypic platforms explored influence of this transporter holistically throughout the plant.

**Abbreviations**
ABC: ATP‐binding cassette
AUX/LAX: AUXIN1/LIKE‐AUX1
BLUP: best linear unbiased predictions
CRISPR: clustered regularly interspaced short palindromic repeats
DAS: days after sowing
EGT: *ENHANCED GRAVITROPISM*
LMM: linear mixed model
MDR: multi‐drug resistance
PGP: P‐glycoprotein
PIN: *PIN‐FORMED*
PM: plasma membrane
REML: restricted maximum likelihood
RGA: root growth angle
RSA: root system architecture
RSRatio: root:shoot ratio
WT: wild type

## 1 INTRODUCTION

The uptake of water and nutrients from the soil is critical to support crop growth and productivity. With a rapidly growing global population and the increasing effects of climate change, there is a greater demand for crops that can produce more grain with less resources. Breeding crops with enhanced sustainability relies on access to genetic variation for morphological and physiological traits that influence resource‐use efficiency and adaptation to abiotic stress (Cronin et al., 2007; Vanhala et al., 2004). While there is still limited understanding of the genetics modulating crop development, significant exploration has been performed in model species Arabidopsis, which provides a powerful resource bank of candidate genes to surgically explore the various mechanisms of crop physiology and development. So far, the crop research community has made good progress to investigate the translation of gene targets identified in model species for aboveground architecture (Ahmar et al., 2024; Huang et al., 2021), leaving many promising targets for root system architecture (RSA) as a novel opportunity to improve nutrient‐ and water‐use efficiency. RSA has important functional implications as it is the primary interface for soil nutrient and water acquisition, along with the physical anchoring of the plant and providing resilience to lodging (Bishopp & Lynch, 2015). Among the most important determinants of RSA is the root growth angle (RGA), which is the angle at which the roots grow into the soil.

A key mechanism influencing RGA is how the roots respond to gravity, where a stronger gravitropic response elicits a steeper rooting angle and is often associated with improved drought adaptation through improved access to deep‐soil water (Ho et al., 2004; Kitomi et al., 2020). Root gravitropism is regulated by the phytohormone auxin, a ubiquitous and important chemical responsible for translating external environmental changes into a physiological response by the plant, allowing for rapid adaptation to its environment (Blilou et al., 2005). This process is maintained by polar auxin transporters, establishing asymmetrical organ‐specific and organism‐wide concentration gradients that regulate cell elongation to enable root growth along the gravity vector. Removing the root cap through genetic ablation results in loss of gravity sensing in the roots and increased lateral rooting, a response consistent with auxin insensitivity or inhibition (Mirza et al., 1984; Tsugeki & Fedoroff, 1999). The starch‐statolith hypothesis is the prevailing theory on how the root cells sense gravity, where dense starch granules named amyloplasts sediment on one side of the cell aligned with the gravity vector, triggering a signaling cascade (Stanga et al., 2011). While still poorly understood, it is thought that this cascade involves the rearrangement of auxin transporters to reestablish the auxin maximum at the root tip.

Auxin distribution is facilitated by three major classes of transporters: the PIN‐FORMED (PIN) efflux carrier proteins, the ATP‐binding cassette (ABC)‐B/multi‐drug resistance/P‐glycoprotein (ABCB/MDR/PGP) subfamily of ABC transporters, and the AUXIN1/LIKE‐AUX1 (AUX/LAX) influx carrier proteins (Yue et al., 2015). The ABCD/MDR/PGP transporters are a large family of transporters with diverse roles in the transport of a variety of substrates, where some studies have demonstrated their involvement in both influx and efflux with auxin (Geisler & Murphy, 2006). Members of the AUX/LAX family are polarly localized in expressing cells where they facilitate auxin entry into cells through passive diffusion, resulting in unidirectional distribution of auxin due to their asymmetric positioning on the plasma membrane (PM) (Swarup et al., 2001). The most well‐described polar auxin efflux transporters are the PIN proteins, whose polarity is credited as the key determinant of auxin transport direction (Blilou et al., 2005).

There are two main types of *PIN* genes: the “short” PINs and “long” PINs based on the size of the intracellular hydrophilic loop domain that separates the flanking transmembrane domains (Luschnig & Vert, 2014). Short PINs play an essential role in maintaining intracellular auxin concentrations by regulating transport between the endoplasmic reticulum and cytosol (Geisler & Murphy, 2006), whereas long PINs regulate the efflux of auxin out of the cell through asymmetrical localization on the PM. This localization changes in response to environmental stimuli by cycling the receptors between the PM and endosomal compartments (Kirschner et al., 2018). This is established through unique and regulated polar localization of these transporters along the PM, allowing directional transport of auxin and forming unique local distributions for organ‐specific and organism‐wide responses (Blilou et al., 2005). Arabidopsis has a total of nine known PIN transporters, whereas higher order species such as cereals often have an increased number of PIN transporters. For example, barley has 13 putative PIN transporters with Hv‐PIN1 and Hv‐PIN2 being homologous to Arabidopsis long PINs At‐PIN1 and At‐PIN2. In contrast, Hv‐PIN5, Hv‐PIN8, and Hv‐PIN9 exhibit short hydrophilic regions. While monocots do not encode At‐PIN3, At‐PIN4, and At‐PIN7 homologues, barley uniquely has four Hv‐PIN10s, which display atypical structure of the hydrophilic loop being noncentral (Hv‐PIN10b2) or missing entirely (Hv‐PIN10a, Hv‐PIN10b1, and Hv‐PIN10b3) (Kirschner et al., 2018).

The developmental plasticity of plants is underpinned by direct and indirect effects. Direct effects controlling organ formation are primarily the consequence of local hormone interactions. Indirect effects can be seen in the dynamic balance of allometry where growth of one organ influences another. For example, the canopy has an allometric relationship with the root system, where increases in the canopy biomass correlate with increased root biomass (Borrell et al., 2022). As *PIN2* expression is almost exclusively within the roots, the possibility of root‐specific architectural changes, independent of aboveground and belowground allometry, provides an interesting subject for investigation (Müller et al., 1998). PIN2 is a known regulator of root gravitropism; it localizes to the shootward side of epidermal cells and mediates the auxin flow from the root tip toward the elongation zone where the auxin response is activated and inhibits cell elongation (Casimiro et al., 2001; Kleine‐Vehn et al., 2008). In *Arabidopsis*, *pin2* mutants show defective root gravitropism because of the mutants’ inability to accumulate auxin in the roots, a loss of curving in RSA formation.

While the PIN2 pathway is well‐studied in model species, knowledge of the extent to which *PIN2* function is preserved across species, particularly monocots, is lacking. In this study, we explore the function and potential of targeted manipulation of the *PIN2* orthologue (Hv*‐PIN2*) in the major cereal crop, barley (*Hordeum vulgare*). We created a knockout in barley using CRISPR/Cas9 (where CRISPR is clustered regularly interspaced short palindromic repeats) genome editing and adopted a holistic plant phenotyping approach that measured a range of root and shoot traits across growth stages to provide new insights into the function and potential utility of genome editing to support crop improvement.

**Core Ideas**

- *PIN‐FORMED* (PIN2) regulates root system architecture in barley.
- Gene editing was used to create novel genetic variants.
- Outcomes show wider root systems without impacting canopy development.
- In‐depth root phenotypic platforms explored influence of this transporter holistically throughout the plant.

## 2 MATERIALS AND METHODS

### 2.1 CRISPR/Cas9 editing of *PIN2* in barley

Genome editing of *PIN2* (*HORVU7Hr1G110470*) in barley was performed using cv. Golden Promise via particle bombardment following the tissue culture protocol described in Harwood et al. (2009), with modifications and gene editing strategy explained in Massel et al. (2022). Wild‐type (WT) embryos that did not go through transformation remained on nonselective regeneration media. Regenerants that produced shoots at least 2 cm long were subsequently transferred to rooting media until development of adequate roots before sowing in glasshouse.

Methods for selection of gRNA are described in Massel et al. (2022). Using those parameters, two gRNAs (G1: 5′‐GTGGCCAACAAGTTCAAGGG‐3′, G2: 5′‐GCCGGTAGTCCATGGCGTAG‐3′) targeting coding regions of the *PIN2* orthologue were selected and cloned into the plasmid system. The gRNA target sequences were placed within the NPTII selection vector for co‐bombardment alongside a plasmid containing zCas9 under the maize ubiquitin promoter. Following isolation of barley immature embryos, transformation was performed via microprojectile co‐bombardment using biolistic techniques (Liu & Godwin, 2012) using the particle inflow gun delivery system with modifications to optimize the system for barley where the vacuum pressure remained at −90 kHa with helium being shot at a pressure of 800 kPa (Massel et al., 2022). Immature embryos were bombarded approximately 5–7 days post isolation with approximately 25–30 embryos per shot before being placed on callus induction medium.

Following successful tissue culture, leaf tissue samples were collected from the putatively edited lines for DNA extraction, polymerase chain reaction (PCR), and DNA Sanger sequencing to identify the nature of the edit. PCR was performed using MyTaq (Bioline) DNA Polymerase in reactions containing 30 ng of DNA template, 1X MyTaq Buffer, 0.5uM of the forward and reverse primers (Table 1) and run following the manufacturer's instructions. PCR reactions were run on 1.2% tris‐acetate‐EDTA agarose gel with a standard 1 kb ladder (New England BioLabs) to confirm formation of products. PCR clean‐up was performed with ExoSAP‐IT (Thermo Fisher Scientific) before being sent for Sanger sequencing. CRISPR analysis tool Synthego (Synthego ICE Analysis tool v3; Synthego, 2024), along with sequence alignment tools, were used to determine types of gene edit. Transmembrane prediction software TMHMM was used to estimate resultant impact on protein function (Krogh et al., 2001).

**TABLE 1:** Primers used in PCR to detect presence of gene and potential edits following transformation.

Target gene	Forward/ reverse	5′ Top 3′ sequence	Tm (°C)	Ta (°C)	Product size (bp)
Hv‐*PIN2*	Forward	GCGTTGGTGGGGCATCTTCA	61	62	1116
	Reverse	TGTGCAGCTCCTTGTTCGAGT	62		

Abbreviations: PCR, polymerase chain reaction; PIN, *PIN‐FORMED*; Ta, temperature of annealing; Tm, temperature of melting.

### 2.2 Phenotyping seminal root angle

The “clear pot” method, routinely applied in wheat and barley root research (Richard et al., 2015; Robinson et al., 2016), was used to evaluate the *pin2* knockout and WT barley lines for seminal root angle at the seedling stage. Seminal root angle is considered a useful proxy trait that can be assayed in a high‐throughput manner and is often associated with the root angle of mature plants in the field (Alahmad et al., 2019; Voss‐Fels et al., 2018). Plants were watered daily and housed in a temperature‐controlled PC2 glasshouse, with 22°C /17°C (day/night) temperature cycle and an 18h/6h (day/night) photoperiod with 450 nm and 660 nm 5700K white LED growth lights.

**FIGURE 1:**
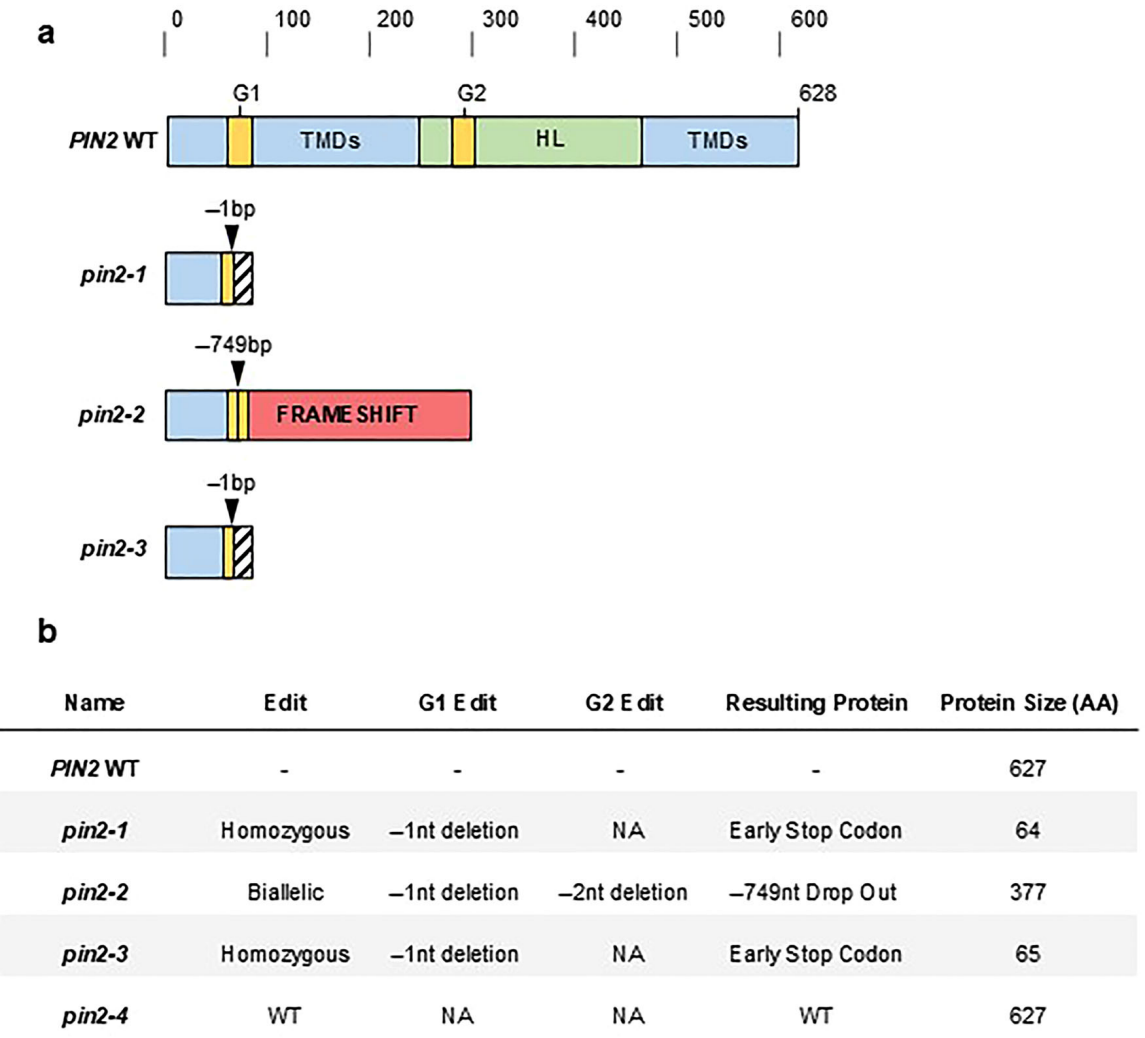
Summary of barley *pin2* knock‐out lines created using genome editing and resultant protein structure changes. (a) Map of alleles for the long‐looped *PIN‐FORMED* (*PIN*) gene showing generalized edits to protein sequence. Scale represents the number of amino acids. Specifically, *pin2‐1* displayed an early stop codon following G1, *pin2‐2* displayed a 749 bp drop out along with a frame shift for the remaining coding region, and *pin2‐3* showed the same editing event‐1 nt deletion at G1 and was homozygous. Notably, *pin2‐4* displayed no genome editing at *PIN2* and was treated as the tissue culture (TC) control for future experiments. (b) Outcomes table of transgenics, with the number of alleles edited at each guide location and the resulting PIN2 protein. G1 and G2, guide sites one and two for CRISPR/Cas9 (where CRISPR is clustered regularly interspaced short palindromic repeats) genome editing; HL, hydrophilic loop; TMD, transmembrane domain. NA indicates no change to sequence.

All edited lines were assessed and compared to the WT as well as a tissue culture control plant (i.e., unedited line regenerated from tissue culture). The edited lines screened included two mutants with varied edits (*pin2‐1* and *pin2‐2*, Figure 1). Seeds were sown in 4‐L ANOVApot of 200 mm diameter and 190 mm in height using a randomized design ensuring 12 reps for each line, with a maximum of 24 seeds per pot. Seeds were sown at a depth of 2 cm at a 45° angle, with the embryonic axis of each seed oriented downward, every 2.5 cm along the transparent wall of the pot to result in 24 barley seeds per pot (Robinson et al., 2016). UQ‐23 potting mix was used, containing 70% composted pine bark 0–5 mm and 30% cocoa peat.

The pots were thoroughly watered the day before sowing, and no additional water was provided, which ensured the orientation of seeds did not shift during germination. After sowing, each clear pot was placed inside a black pot of the same dimension to prevent light from influencing root development. Images of roots were captured at both 3 and 5 days after sowing (DAS) using a camera (iPhone 13 Pro Max). Analysis for seminal root angle and number was determined using ImageJ (Schneider et al., 2012), measuring the average deviation angle of the first pair of seminal roots to the vertical plane (Christopher et al., 2013; Richard et al., 2015; Robinson et al., 2016).

### 2.3 Evaluating gravitropic response

To visualize and monitor the gravitropic response, *pin2* seeds (*pin2‐1* and *pin2‐2*) were germinated in glass chambers (210 mm × 297 mm) lined with black serviettes that were watered and rolled flat to remove air bubbles prior to placing seeds. The developing plants were grown in darkness at 25°C. After 4 days, images of the roots were captured and then chambers were rotated by 90°. Root images were captured at 0, 4, 6, 8, and 24 h after rotation, with the root position marked at each time point. The responsiveness of the root system to gravity was quantified by measuring the change in angle of the root tips for seminal roots and the radicle over time (Kirschner et al., 2021).

For data analysis, the root angle values for the radicle root tip were measured in relation to the horizontal, with the angle following rotation set to 0°. All root angle values were obtained with the software ImageJ (Schneider et al., 2012). The average root angle at each time point and change in root angle over time were calculated and compared using a two‐tailed *t*‐test.

### 2.4 Evaluating root system architecture in rhizoboxes

The RSA of the *pin2* mutants and WT lines were assessed in a series of rhizobox experiments. RSA was assessed by determining the root angle, total biomass, shoot biomass, root biomass, and root density at various depths. This set‐up uses wooden boxes lined with a plastic sheet and placed upright on a bench with capillary matting at the base of the bench laid out in a randomized complete block design. The first two experiments were conducted using the rhizobox platform described by Kang et al. (2024) on *pin2‐2* and WT. The chambers were 30 cm (width) × 5 cm (thickness) × 80 cm (depth) and were filled with potting mix containing 6 g/L of Osmocote mixed in thoroughly. Plants were watered daily and housed in a temperature‐controlled PC2 glasshouse, with 22°C/17°C (day/night) temperature cycle and standard long‐day conditions using a 18h/6h (light/dark) photoperiod. Two different planting densities were tested, first with three plants per chamber and second with one plant per chamber. In the first experiment, six to nine seeds were evenly dispersed throughout the width of the chamber near the surface of the potting mix (5‐cm depth). After emergence, each chamber was thinned down to three plants per box. In the second rhizobox experiment, a total of three seeds were sown (depth of 5 cm) per rhizobox with one plant retained after emergence. The experiments in these rhizoboxes were harvested after 4 weeks, as the root systems had almost reached the base of the chambers. Two days prior to harvesting, the plants were heavily watered and were not watered the day prior to harvest. Upon harvesting, in both rhizobox experiments, each chamber was divided into three equal segments (26‐cm depth) to represent “upper,” “middle,” and “deep” layers. The potting media‐containing roots sampled from each section were collected and bagged individually. Each root segment was hand washed to remove potting mix, placed in a paper bag, and dried at 80°C before weighing.

While the plants grew well in the 30 cm width boxes, the narrow width could not accommodate the outgrowth of the mutant root system as the roots reached the sides of the chamber early in the plant growth cycle, resulting in either “training” of the roots downward or “bouncing” off the wall and back into the substrate. This reduced the ability to accurately quantify differences in RSA. To accommodate the wider root system, “double width” rhizoboxes (60 cm × 5 cm × 80 cm) were constructed to improve the visualization of the entire RSA.

In the double width rhizboxes, plants were phenotyped after 42 days of growth, which enabled the analysis of root systems that were unimpeded and more extensively developed. The same potting mix, growing, and plant maintenance parameters were used and included assessing both *pin2‐1* and *pin2‐2* compared to the WT to validate the trait. Two trials were performed using the double width rhizoboxes. The first included six replicates each for *pin2‐*2 and WT, and the second trial consisted of four replicates each for *pin2‐2, pin2‐1*, and WT. For the double width rhizobox experiment, a total of three seeds were sown (depth of 5 cm), which were later thinned to one plant per rhizobox. Thus, it was anticipated that growing barley plants in these wider rhizoboxes would provide a clearer visualization of the root system branching and distribution down to a depth of 80 cm and root:shoot biomass partitioning in a setting that more closely resembled field conditions. The double width rhizobox was analyzed by separating the chamber and image into nine biomass sections (3 × 3 sections of 20 cm × 26 cm). As the root structures of the individual plants in the wide rhizoboxes could not be separated, the root biomass measurements represented the total root biomass produced by the three plants.

To synchronize germination, a cold treatment was performed by placing the seed in a Petri dish lined with a moist paper towel overnight at 4°C, then plates were moved to room temperature (22°C) for 24 h prior to sowing. The rhizoboxes were well‐watered daily by manually adding water to the top until drainage was observed from the bottom. During the experiment, a leaf sample from each plant in the box was taken for DNA extraction to confirm the genotype of the plants.

The root system was photographed for image analysis, first converted into a black and white image to be uploaded into RhizoVision Explorer. This software was used to quantify proportion of roots with shallow, medium, and steep root angles grouping by those <30°, <60°, and <90° from the medial axis (Seethepalli et al., 2020). The suggested default parameters for “whole root” analysis were used, with an image thresholding level of 120, an edge smoothing threshold of 2, and root pruning threshold of 1. To investigate potential changes in aboveground biomass, the entire shoot sample for each plant was removed and placed in individually labeled bags.

### 2.5 Evaluating aboveground traits at physiological maturity

To further explore any shoot trade‐offs associated with *pin2* in barley, both the *pin2‐1* and *pin2‐2* edited lines alongside the WT were grown under controlled conditions and evaluated at physiological maturity. Plants were sown in 1‐L pots at a density of one plant per pot using eight reps per genotype arranged in a randomized complete block design. Plants were grown under stress‐free conditions using a temperature regime of 22°C/17°C (day/night) and a standard long‐day photoperiod of 18h/6h (light/dark). At maturity, each plant was partitioned into spikes, stems with leaves, and grains. Plant height, spike length, and the number of seeds per spike were recorded for the primary tiller of each plant. The total number of fertile tillers (grain‐producing tillers) and days to flowering were recorded for each plant.

### 2.6 Analysis of root and shoot phenotype data

To analyze data from the gravitropism experiment, the root angles obtained by *pin2* edited line and WT line were compared using parametric *t*‐tests and one‐way analysis of variances with multiple comparisons where *H*_0_ > 0.05 using GraphPad Prism (v9.3.1).

All root and shoot data collected in glasshouse phenotyping experiments, including clear pot, rhizobox, and maturity studies, were analyzed using a linear mixed model (LMM) to account for spatial variation and maximize the genetic variance captured in each trait‐specific model. The LMM was fitted with the following equation:

y=Xτ+Zu+ε,

where *X* and *Z* are design matrices associated with fixed (τ) and random (u) effects and ε is the residual error. A custom LMM was fit to each trait measured across the glasshouse root and shoot experiments, with the best model fit determined by the Wald test statistics (for fixed effect terms only), the highest restricted maximum likelihood (REML) loglikelihood value, and the smallest Akaike information criterion (Akaike, 1974; Kenward & Roger, 1997). The specific design terms fitted as random and fixed effects in the LMM for each trait are detailed in Table S1. The auto‐regressive residual variance structure in the order of 1 was found to fit best across all traits and was thus fitted across all trait LMMs. A generalized measure of broad‐sense heritability was calculated for each trait to quantify the proportion of phenotypic variance that is attributed to genetic effects. This was determined using the following equation:

$$h_{g}^{2}=1-\frac{A_{tt}}{2\gamma_{v}},$$

where $A_{tt}\;$ is the mean predicted error variance and $\gamma_{v}\;$ is the genetic variance (Cullis et al., 2006). The LMMs were fit using ASReml‐R in the R software environment (Posit Team, 2025), and best linear unbiased predictions (BLUPs) were calculated from the variance components of the model (Butler et al., 2017). Figures displaying and comparing trait BLUPs between *pin2* edited lines and the WT were created using the ggplot package in R (Wickham et al., 2016).

## 3 RESULTS

### 3.1 Gene edited *pin2* knockout lines in barley

A CRISPR/Cas9 strategy was used to generate several mutant alleles in the barley *PIN2* gene. The *PIN2* orthologue in barley was identified using Phytozome, showing 92.7% and 92.5% protein homology to *Sorghum bicolor* (Sb‐*PIN11*) and *Oryza sativa* (Os‐*PIN2*) respectively, with both being orthologues of At‐*PIN2* (Goodstein et al., 2012). Four stable transgenics were created that contained both the bombarded plasmids showing a range of gene editing outcomes (Figure 1). Of these, two lines were shown to contain edits in at least one of the target sites. Sanger sequencing was used to verify the editing outcomes, where the first showed a homozygous‐1 nt deletion, resulting in a frameshift of the coding region. This leads to a truncated peptide fragment that only contains 64/627 of the WT protein sequence before stopping and will be referred to as *pin2‐1*. The second mutant was biallelic and edited at both sites and was biallelic where one allele was a −749 bp deletion in the sequence between the two guide sites resulting in a frameshift mutation with 65/627 of the original peptides and 377 amino acids before stopping, while the other allele only had the G1 edit causing an early stop codon. The homozygous large deletion will be referred to as *pin2‐2*, whereas segregation for the homozygous mutant resulted in *pin2‐3*, an edited line with an early stop codon producing a peptide 65/627 of the original. Lastly, one line showed no evidence of editing at target sites and was described as *pin2‐4*. Editing efficiency was approximately 75% at G1 and 25% at G2. Transmembrane prediction using TMHMM displayed a loss of the transmembrane domains before the central hydrophilic loop, leading to complete loss of essential domains for the function of PIN proteins as an auxin efflux carrier (Figure S1).

### 3.2 Loss of PIN2 produced altered root growth angle

**FIGURE 2:**
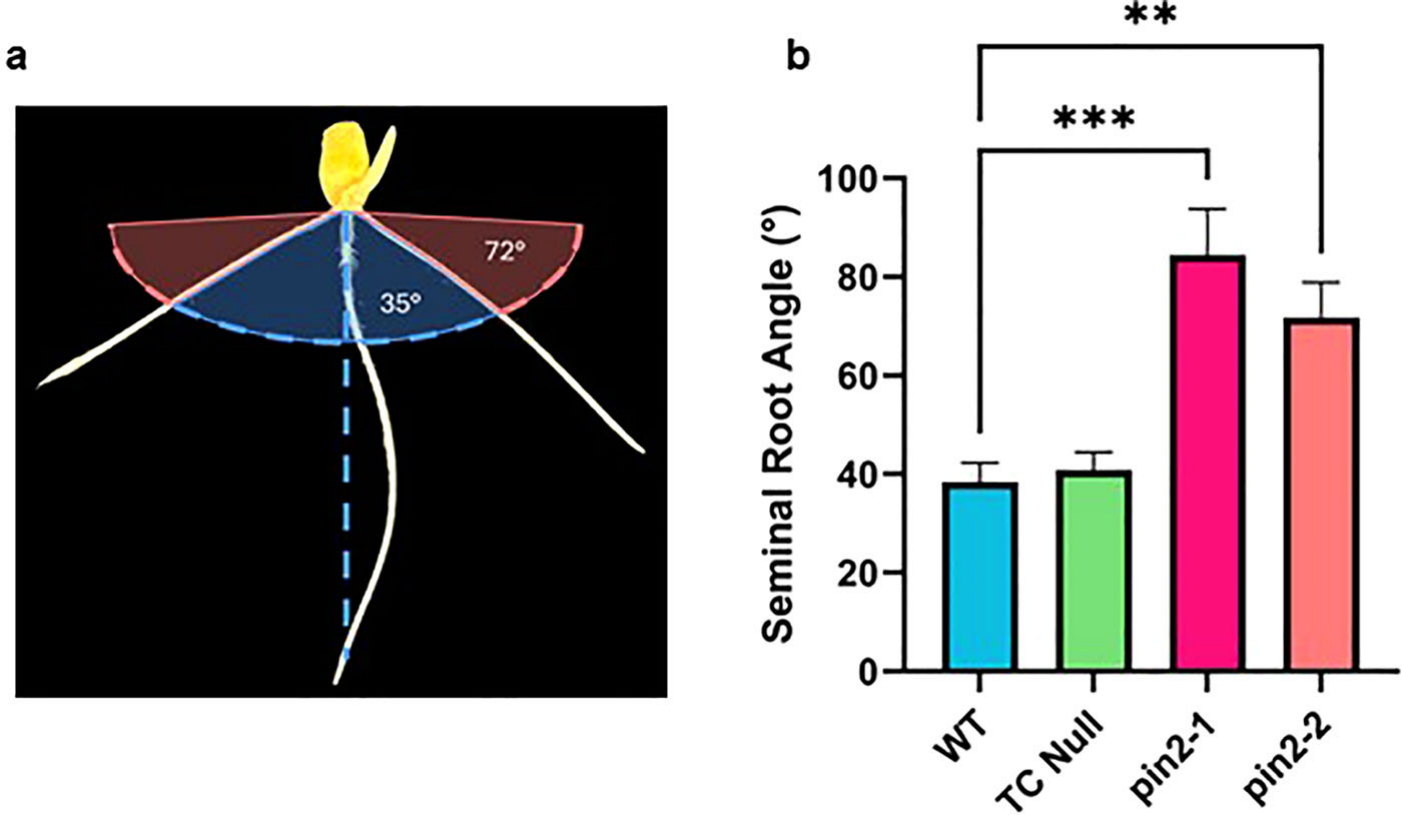
Barley *pin2* knock‐out lines display significantly wider seminal root angle. (a) Representative image of wild type (WT) seminal root angle illustrating how the measurements for seminal root angle were recorded in this study with representative image of WT compared to *pin2‐2* (pink). Measurements were recorded in ImageJ for the first pair of seminal roots to the vertical axis and the two angles summed to give a representative measurement of root angle. (b) Comparison of seminal root angle phenotypes for Golden Promise WT carrying *PIN‐FORMED2* (*PIN2*), tissue culture (TC), null (*pin2‐4*), and transgenic knock‐out lines for *pin2* (*pin2‐1* and *pin2‐2*).

To determine if Hv‐*PIN2* plays a role in root growth orientation, the seminal root angle was evaluated using the clear pot system. This was performed on two homozygous knockout lines, *pin2‐1* (*n* = 9) and *pin2‐2* (*n* = 7), alongside a tissue culture null (*n* = 11) and WT (*n* = 16) as controls. The seedlings were imaged at days 3, 5, and 12. Both *pin2‐1* and *pin2‐2* displayed a significantly wider seminal root angle, from 38° in WT to 85° and 72° between *pin2‐1* and *pin2‐2*, respectively, displaying similar patterns of agravitropism as those seen in the previous gravitropism assay, where roots appear “aimless,” growing in the direction that they emerged (Figure 2b).

### 3.3 *PIN2* controls root gravitropism in barley

**FIGURE 3:**
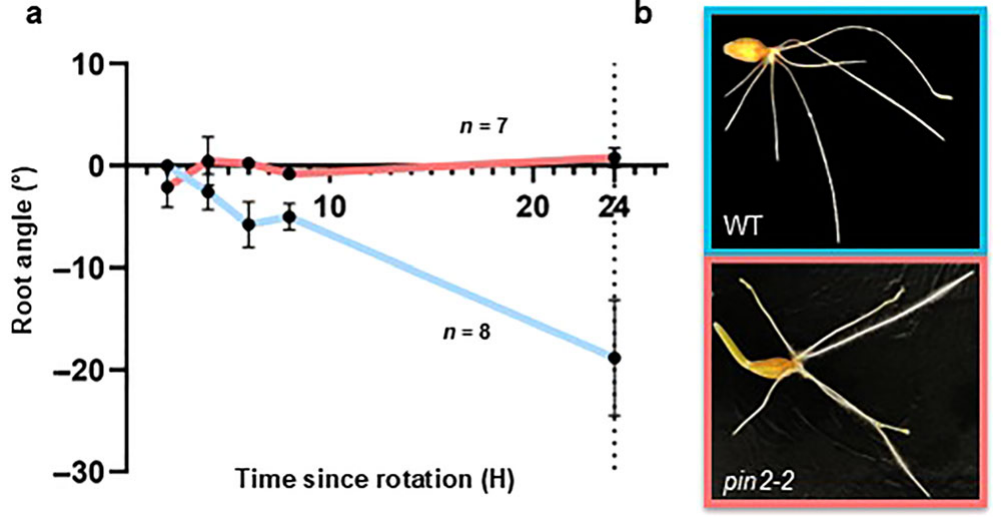
Barley *pin2* knock‐out lines display root‐specific agravitropism at the seedling stage. (a) Change in root angle for *pin2* knock‐out lines (*pin2‐1* and *pin2‐2*) (red) and wild type (WT) (blue) over 24 h from seedling gravitropic assay conducted in glass panels. Root angle was measured from the radicle in relation to the horizontal axis, and measurements were taken every 2 h for the first 8 h, then again after 24 h (*n* = 7). The derivative of root angle over time was taken as a standardized measurement for strength of gravitropic response. Bars show standard error of the mean change in seminal root angle over time. (b) Images of WT and *pin2* seedlings captured 24 h after rotation.

To evaluate the mechanism behind the increased root angle in *pin2* mutants, root gravitropism assays were performed. Plates carrying seedlings were rotated 90° following germination to gravistimulate the roots and visualized over the course of 24 h. The rate of change in root angle over time is representative of both gravitropic response and strength of its response. The roots of *pin2‐2* (*n* = 7) lacked a gravitropic response, where the roots continued to grow from their initial sprouting (Figure 3A). On the other hand, WT (*n* = 8) roots showed a linear rate of change to root angle toward the gravity vector at an average of 0.83°/h. The shoots appeared to respond to gravity, with both WT and *pin2‐2* curving upward following rotation (Figure 3B, Table S3).

### 3.4 Barley *pin2* knockout lines display wider and shallower root architecture

To explore the role of *PIN2* on RSA, a series of rhizobox experiments were conducted, which compared *pin2‐1* and the WT. Initial phenotyping was conducted using narrow rhizoboxes at two planting densities: one plant per box (15 cm row spacing) and three plants per box (5 cm row spacing) at 28 DAS. In the lower density experiment, results revealed that *pin2‐1* produced approximately 40% more root biomass in the upper section (S1) of the rhizoboxes compared to the WT (*p* < 0.05, Figure S2c). No difference in shoot biomass or total biomass was detected (*p* > 0.1, Figure S2a).

In the high‐density experiment, *pin2‐1* showed a reduction in total root biomass of approximately 30% (*p* = 0.07, Figure S2a). Again, there was no change to total aboveground biomass between samples (*p* > 0.1). The major difference in root distribution was observed in the lower section (S3), where *pin2‐1* produced significantly less root biomass in both low‐ and high‐density experiments (Figure S2). As a result of the reduction in root biomass, *pin2‐1* showed a significantly lower root:shoot ratio (RSRatio) compared to the WT at 1‐planting density and trending reduction at 3‐planting density (*p* < 0.001, *p* = 0.057).

**FIGURE 4:**
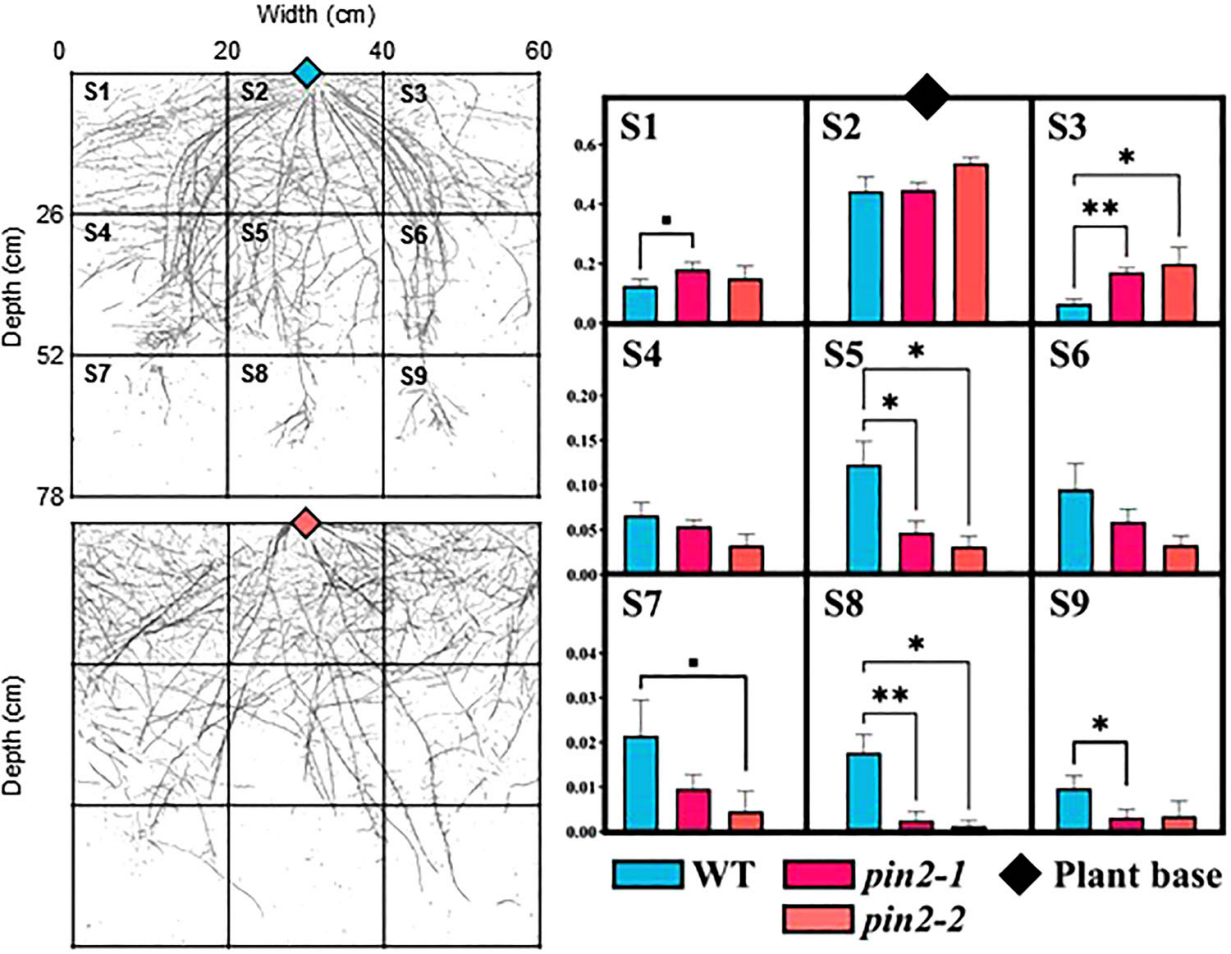
PIN‐FORMED2 (PIN2) in barley regulates downward root growth leading to steeper and narrower root architecture. Spatial root system configuration of the wild type (WT) and *pin2* barley lines (*pin2‐1* and *pin2‐2*) in the wide rhizobox chambers. Left images show representative images of the root systems for both the WT (blue) and *pin2‐1* (red). Solid lines indicate each section (S1–S9). The mean ratio of dry root biomass produced in each section to total root biomass is displayed as bar charts on the right. Significance was calculated by nonparametric *t*‐test. *N* = 10 for *pin2‐1* and WT. *N* = 4 for *pin2‐2*. *p < 0.05, **p < 0.01.

It was evident that the narrow width of the rhizoboxes in the first set of experiments constrained our ability to accurately phenotype RSA of the *pin2* mutants as the roots hit the sides and were trained downward (Figure S2b). Thus, rhizoboxes with double the width were used to subsequently phenotype the lines in a scenario where root growth was less impeded. In the wider rhizoboxes, *pin2‐1* and *pin2‐2* exhibited a wider and shallower root system, as demonstrated by the increased root biomass produced in the upper wide sections (S1 and S3; *p* < 0.1 and *p* < 0.05, respectively, Figure 4). The upper middle section (S2) (i.e., directly below the base of the plant) showed no significant change in root biomass, whereas the middle section (S5) showed a significant reduction in root biomass for both lines (*p* < 0.05). In the wide rhizoboxes there was no significant change to aboveground (*p* > 0.1) or belowground biomass (*p* > 0.1) in *pin2‐1* and *pin2‐2* compared to the WT. However, image analysis of the whole root system using RhizoVision Explorer showed that *pin2‐1 and pin2‐2* displayed a higher proportion of roots classified as “shallow” (*p* < 0.05), and a significant increase in biomass in the upper sections relative to WT, indicative of shallow rooting behavior (Figure S3).

### 3.5 *PIN2* has a limited role in aboveground development

Canopy traits were assessed during the rhizobox studies to explore the allometric relationships or potential aboveground trade‐offs in these lines. Across both rhizobox trials, no significant difference in canopy biomass was detected despite the significantly reduced root biomass. Early vigor measurements conducted on the three plants per rhizobox trial revealed no significant difference in tiller number (*p* > 0.1) or leaves per tiller (*p* > 0.1). Tiller number was approximately 15 across WT and *pin2‐1* with an average of five leaves per tiller (Figure S3). Similarly, leaf size was consistent across mutant and WT (Figure S3). The root:shoot biomass ratio was significantly altered in the *pin2* mutant from 0.7 in WT to 0.5 at higher density (5 cm) row spacing and from 0.5 in WT to 0.4 in *pin2* at lower density (15 cm) row spacing, representing a general reduction of root biomass of 20% (*p* < 0.1, *p* < 0.01, Figure S2a,c).

A maturity study was conducted (*N* = 8) to explore canopy traits at maturity, where no significant changes to aboveground canopy biomass, number of fertile tillers at maturity, and days to flowering were observed (*p* > 0.05). Following spatial analysis, no genotypic effects were associated with these traits between WT and either of the *pin2* mutants (Figure S4).

## 4 DISCUSSION

### 4.1 *PIN2* is essential for root gravitropism and root angle

The spatial and temporal distribution of roots via branching, elongation, and curving all affect the ability of the crop to extract water and nutrients from the soil (Manschadi et al., 2006; Rich & Watt, 2013). A substantial body of research suggests that optimized RSA can improve water acquisition with minimal metabolic cost, potentially improving crop yields under both favorable and adverse conditions (Uga, 2021). Specifically, variation in gravitropism can affect RGA, altering root anchoring and exploration of different soil layers to acquire water and nutrients, as shown for *ENHANCED GRAVITROPISM 2* (*EGT2*) in barley and wheat (Kirschner et al., 2021). However, our knowledge of all the genes involved in orchestrating root growth and gravitropism is limited, particularly in cereals.

The identification of auxin‐related mutants in model species like Arabidopsis has played a key role in better understanding how auxin orchestrates plant morphogenesis. For example, loss of At‐*PIN2* causes root gravitropism defects due to the mutant's inability to transport auxin from the roots toward the shoots, and, due to *PIN2*'s high levels of conservation in cereals, a similar function is predicted in cereals that exhibit more complex root architecture (Lu et al., 2015). Here, we report that *PIN2* plays an important role in gravitropism and root angle in barley. The loss of *PIN2* causes root‐specific agravitropism that led to increased seminal root angle resulting in wider and shallower rooting, without impacting the aboveground traits. These findings demonstrate the potential for *PIN2* as a new target to improve climate resilience while maintaining the agronomic traits of the canopy that have already been optimized through breeding for different crop production regions.

Deep root systems can contribute to drought adaptation by improving access to stored soil moisture in deep soil layers, thereby providing an advantage during terminal or post‐flowering drought stress. The root system of the crop can exhibit morphological, structural, and physiological responses to changes in the growing environment, referred to as root developmental plasticity. This is underpinned by changes in root branching, elongation, and curving, which broadly define the extent to which the root system can explore the soil volume. The “curving” component is associated with angle of seminal roots and the seedling proxy trait of mature root angle (Kang et al., 2024).

Despite the more complex genetic and physiological architecture of barley, our analysis of tropic responses through *pin2* knockouts revealed homologous function to that of the model species Arabidopsis (Müller et al., 1998). Typically, gravitropism is a guiding force whereby the radicle grows directly downward, followed by a slight weakening of the response to initiate growth at non‐vertical angles in order to facilitate roots spreading for soil exploration (Sachs, 1882). This is termed the gravitropic setpoint angle and is considered a balance between two antagonistic growth signals, gravitropism and an anti‐gravitropic offset (Digby & Firn, 1995). In barley, the EGT1 and EGT2 genes have been identified as key elements in this antigravitropic response (Fusi et al., 2022). Unlike PIN genes that are responsible for auxin transport, they are involved in cell wall stiffness at the elongation zone, further upstream than PIN2, where mutant EGT1 resulted in steeper root growth (Kirschner et al., 2024). In *pin2* knockouts, the roots appeared aimless, and instead of exhibiting a consistent growth angle in response to gravity as typically seen in seedling assays, the roots continued to grow from their point of initiation out of the soil, against the gravity vector (Figure 3). In addition, phenotyping seedlings for seminal root angle, an established proxy for mature root angle, showed significant increases in seminal root angle (Alahmad et al., 2019; Kang et al., 2024). These observations suggest the loss of gravitropism leads to a loss of the primary guiding force for root angling, one of the earliest drivers of root distribution and architecture formation (Digby & Firn, 1995; Nakamoto & Oyanagi, 1996; Roychoudhry et al., 2013).

There is a well‐established link between root distribution in the soil, water access, and water use (Li et al., 2022; Robertson et al., 1993). Further evidence on the role of *PIN2* and gravitropism in directing RSA formation in barley comes from functional analysis. The increased root angle resulting from inhibited apical auxin transport caused a wider and shallower root system in *pin2*, resulting in larger root biomass in upper layers, while trending toward a reduction in roots at depth, resembling a topsoil foraging ideotype that is beneficial for capturing phosphorous and intermittent rainfall (Lu et al., 2015; Lynch, 2013, 2019).

### 4.2 *PIN2* mutants cause root system‐specific changes

In barley, the quiescent center is a group of approximately 30 cells located in the root tip (apical meristem) of vascular plants where cell division occurs (Kirschner et al., 2017). In Arabidopsis, PIN1 acts in shoot to root auxin transport, transporting auxin toward the quiescent center of the root meristem (Blilou et al., 2005), whereas the PIN2 functions basally of the quiescent center and meristem to inhibit cell elongation in the root tip. Once auxin is transported to the root tip via PIN1, it accumulates at the root meristem where the high auxin concentrations inhibit elongation (Swarup et al., 2005). Subsequently, PIN2 transports auxin shootward along the apical side of the roots, inhibits the roots elongation, and causes the roots to curve downward, also known as the gravitropic response (Blilou et al., 2005). Throughout plant development, auxin synthesized in leaf primordia is continually transported to the root tip and the process is repeated, allowing for gravitropic root elongation. As such, in the barley *pin2* mutants, we hypothesize there is a build‐up of auxin in the lateral root cap post‐quiescent center and cause of the root‐specific changes to cell expansion due to an inability to flush auxin shootward, explaining the limited gravitropism and leading to increases in root angle and causing a visible agravitropic phenotype. Meanwhile, without the shootward flushing of auxin, it pools at the root tip, inhibiting elongation apically and basally due to the lack of polar auxin transport (Figure S5).

Overall, this downstream effect on root elongation allows for more subtle changes to root architecture without significantly affecting organism‐wide patterning and architecture. For example, in maize, the overexpression of Zm‐*PIN1a* showed an elevated concentrations of auxin in the roots, increasing lateral root formation and seminal root length, improving root weight, and shortening plant height (Forestan & Varotto, 2010), whereas based on our findings in barley, *PIN2* has been shown to be a root‐specific auxin efflux transporter required for gravitropic responses in the roots. Loss‐of‐function produced aimless rooting with agravitropism, leading to increased root angle and a shift toward a top‐soil foraging RSA with a potential increase in root biomass (Figure S5).

Interestingly, under the well‐watered conditions used in this study, the *pin2* barley mutant did not show any difference in aboveground biomass or agronomic performance (Figure S3). This is contrasting with the overexpression of *PIN2* homologue in rice, which showed significant changes to plant height, tiller number, and tiller angle, while *pin2* mutants showed no change to canopy branching (tiller number), leaves per tiller, area per leaf, number of fertile tillers, or days to flower (Chen et al., 2012). The lack of changes to canopy architecture or flowering time in cereal crops suggests this root‐specific target could be directly used in the future to breed cultivars with shallow rooting systems. Summarizing, Hv‐*PIN2* has been characterized as the functional homologue of At‐*PIN2*, playing an essential role in root gravitropism and providing new insight into root and shoot auxin transport and its roles in development and gravitropism.

### 4.3 Root:shoot ratio of *pin2* mutants is insensitive to planting density

Genetic and management solutions are required to develop resilient crops in highly variable environments. Many different management systems are possible to combat drought such as row spacing, population, irrigation, fertilization, and cropping systems. Many different genotypic solutions are also possible, including utilizing the *PIN* gene family to modify traits affecting water supply and demand. Understanding the interaction of genotypes with different management strategies for specific target environments is critical to improving yield stability. While *PIN2* clearly plays an important role in orchestrating root curving through gravitropism, it also has novel effects on allometry under different planting densities in glasshouse conditions. Plants with narrow angles and more vertical roots tend to have larger proportions of their roots at depth during later development, which could increase access to water in deep soils. Conversely, genotypes with wider root angle and more horizontal rooting are better able to explore the soil in the inter‐row space, which could increase access to water in skip‐row systems (Hammer et al., 2009).

**FIGURE 5:**
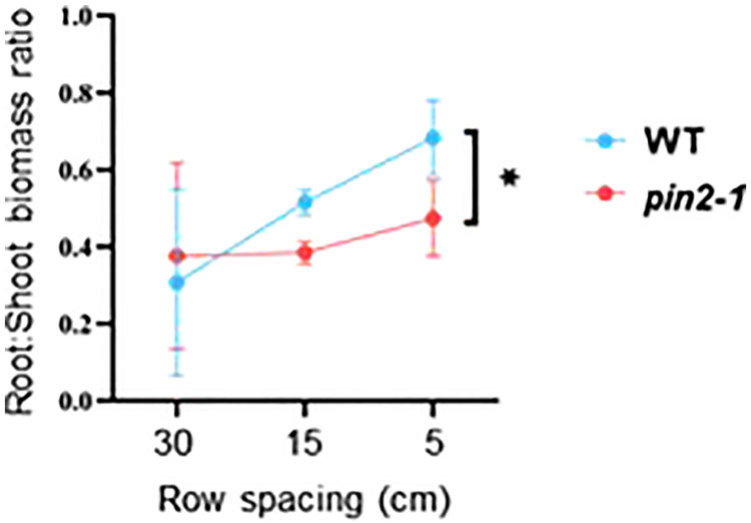
PIN‐FORMED2 (PIN2) increases root biomass under higher planting densities. The root:shoot biomass ratio for wild type (WT) and *pin2‐1* genotypes grown at three different row spacings indicative of plant density. Error bars represent standard error of the mean. Significance was tested using parametric *t*‐test (*p < 0.05).

In fertile environments when water is not limiting, competition among plants in a crop is mainly for light. However, in nutrient‐poor or water‐scarce conditions, competition is mainly for the limiting factor of either nutrients or water, respectively. The effects of competition among root systems are largely determined by the levels of water and nutrient availability, particularly inorganic forms of nutrients such as nitrate and ammonium (Tilman, 1985). The usual row spacing of barley is typically 15–20 cm between rows to maximize the potential of individual plants. In our rhizobox experiments with non‐limiting water and nutrients, planting density (number of plants per rhizobox) was positively correlated with RSRatio in the WT lines. As plants per box increased from 0.5 (30‐cm row spacing) to 1 (15‐cm row spacing) to 3 (5‐cm row spacing), the RSRatio increased from 0.3 to 0.5 to 0.7, respectively (Figure 5). This indicates an increased allocation of resources to the roots as planting density increased, likely a cost in response to increased aboveground competition. Whereas in contrast, the *PIN2* mutant lines maintained an RSRatio of about 0.4 regardless of planting density, highlighting a trending insensitivity of *pin2* mutants to competition that is a reduced or loss of sensitivity to rooting density on root growth (Figure 5). Hence, at higher planting densities there was a significant change in root:shoot biomass ratio, with no significant change to canopy biomass and a reduced root system, consistent with the *pin2* response in Arabidopsis and likely a reflection of the reduction in root elongation (Figure S1; Müller et al., 1998). Field trials will be necessary to fully explore these implications on crop performance across a range of conditions.

In the controlled environment used in the rhizobox experiments, nutrient availability was not a limiting factor. As such, nutrient uptake or the diffusion rate of the ions in the soil may have been limiting factors. While the WT reflexively altered its biomass allocation depending on planting density, increasing belowground biomass as planting density increased, *pin2* maintained a consistent RSRatio at all tested densities (Figure 5). As such, it is hypothesized that *PIN2* plays an important role in external sensing alongside gravitropism, where the absence of this PIN2 transporter leads to agravitropic roots that project outwards and appear insensitive to environmental stimuli such as intraspecific competition. In field‐grown barley, when nutrients are not limiting, less biomass is required underground, and capacity for nutrient uptake can be enhanced by concentrating root proliferation in the topsoil (Grime & Hodgson, 1987; Hecht et al., 2016). It is possible the increased root proliferation in upper soil layers by *pin2* provides improved resource availability, limiting the need for further resource investment into soil exploration. Therefore, modulating the expression of *PIN2* provides a unique target for improving RSA, which may lead to a range of root angles and influence drought tolerance without any negative impacts on yield or obvious aboveground trade‐offs (Joshi et al., 2016). Currently, the complete loss‐of‐function *pin2* mutants display a topsoil foraging root system ideotype that may provide benefits for uptake of immobile nutrients in some agricultural settings. However, field studies in different environments are needed to explore the effects of *PIN2* on yield and its influence under changing planting densities (Liu & Godwin, 2012).

## 5 CONCLUSION

Understanding the allometric relationship between canopy and root architecture has important agricultural implications. Here, we demonstrate conserved function of the auxin efflux carrier Hv‐*PIN2* in directing root angle through the gravitropic response in barley. Alongside altered root distribution that produced a shallower and more expansive RSA, *pin2* mutants did not display the typical root‐shoot allometric relationship. While further investigation is required regarding the exact mechanism regulating root angle, *PIN2* shows clear links to agronomic traits such as root angle and root distribution that may offer an advantage under moisture‐limited environments. The novel root phenotypes identified in this study offer useful insight into the potential for genome engineering of root traits for future barley improvement programs. Overall, the investigation of key developmental genes (initially discovered in model species) through gene editing shows potential to support the development of new cultivars with improved nutrient‐use efficiency and climate resilience.

## AUTHOR CONTRIBUTIONS

**Zachary Aldiss**: Conceptualization; data curation; formal analysis; investigation; methodology; validation; writing—original draft. **Yasmine Lam**: Conceptualization; formal analysis; supervision; writing—review and editing. **Hannah Robinson**: Formal analysis; methodology; writing—review and editing. **Richard Dixon**: Formal analysis; investigation; writing—review and editing. **Laura Steinhardt**: Data curation; formal analysis; writing—review and editing. **Peter Crisp**: Methodology; writing—review and editing. **Ian Godwin**: Conceptualization; funding acquisition; writing—review and editing. **Andrew Borrell**: Funding acquisition; project administration; writing—review and editing. **Lee Hickey**: Funding acquisition; project administration; writing—review and editing. **Karen Massel**: Conceptualization; formal analysis; investigation; methodology; project administration; software; supervision; writing—original draft; writing—review and editing.

## CONFLICT OF INTEREST STATEMENT

The authors declare no conflicts of interest.

## Supporting information




**Figure S1**. Illustration of *PIN2* genome edited knock out lines with impact on protein structure and transmembrane domains compared to wild type.

**Figure S2**. Canopy and root trait results from narrow rhizobox experiments conducted using different planting density.

**Figure S3**. PIN2 in barley regulates downward root growth leading to steeper and narrower root architecture without influencing biomass.

**Figure S4**. Maturity trial reveal no significant difference to canopy and phenology in *pin2*.

**Figure S5**. Summary of hypothesis: disrupted auxin transport in *pin2* genome edited barley inhibits gravitropic response and alters root development.

**Table S1**. Fixed and random effects table for the linear mixed model (LMM) fitted to the trait of interest.

**Table S2**. Synteny of *PIN* genes across cereals.

**Table S3**. Table measured shoot growth angles for WT and *PIN2 KO*.

## Data Availability

All data generated or analyzed during this study are included in this published article and its Supporting Information files.
