# Supplementary figures and images for: The PIN2 ortholog in barley modifies root gravitropism and architecture
Source: Plant Genome. 2025 Aug 6;18(3):e70061. doi: 10.1002/tpg2.70061 (PMC12328839; doi:10.1002/tpg2.70061)

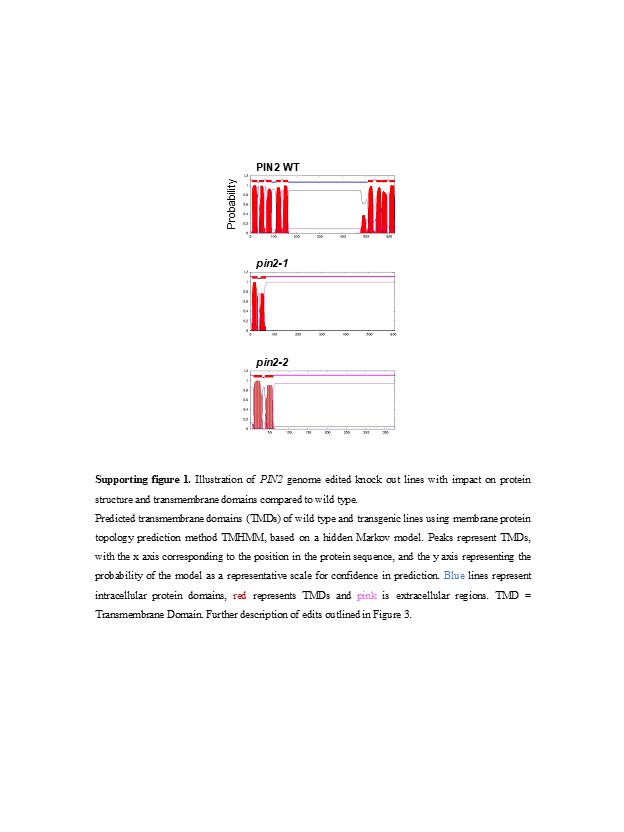

Supplement: Supplementary file 1 — Figure S1. Illustration of PIN2 genome edited knock out lines with impact on protein structure and transmembrane domains compared to wild type. [file TPG2-18-e70061-s001.jpeg]

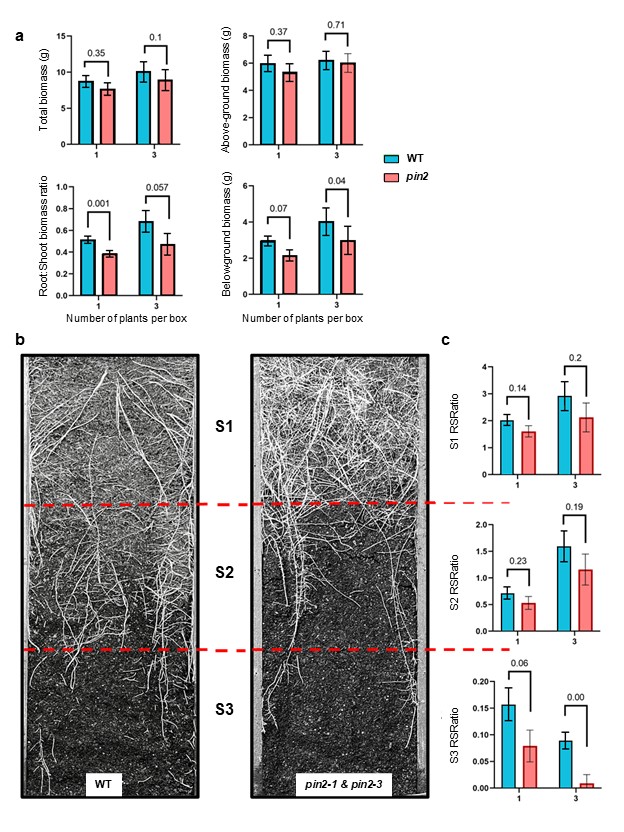

Supplement: Supplementary file 2 — Figure S2. Canopy and root trait results from narrow rhizobox experiments conducted using different planting density. [file TPG2-18-e70061-s006.jpeg]

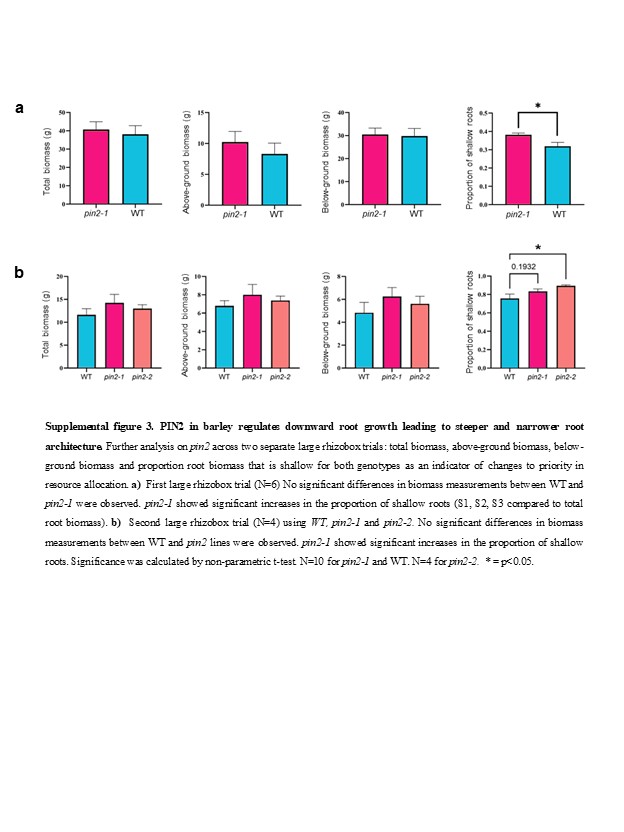

Supplement: Supplementary file 3 — Figure S3. PIN2 in barley regulates downward root growth leading to steeper and narrower root architecture without influencing biomass. [file TPG2-18-e70061-s008.jpeg]

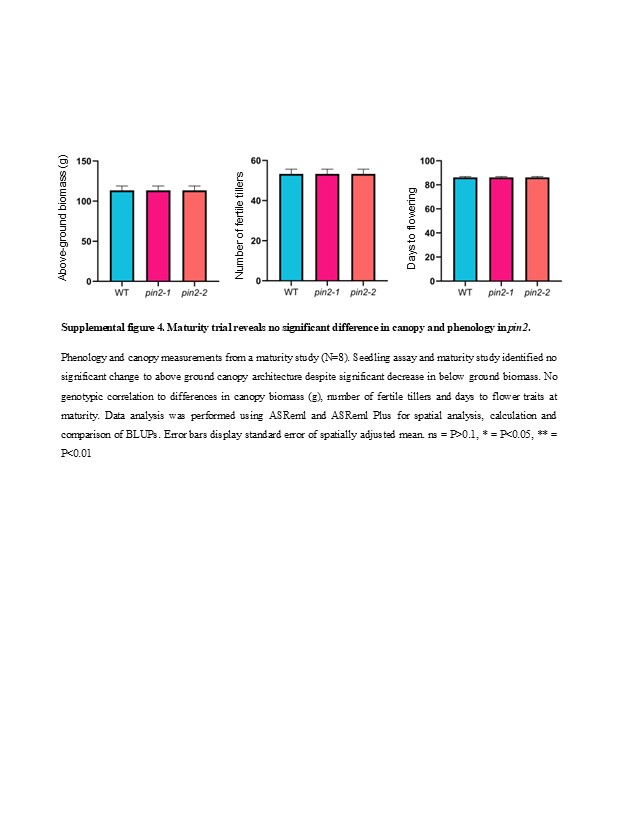

Supplement: Supplementary file 4 — Figure S4. Maturity trial reveal no significant difference to canopy and phenology in pin2. [file TPG2-18-e70061-s004.jpeg]

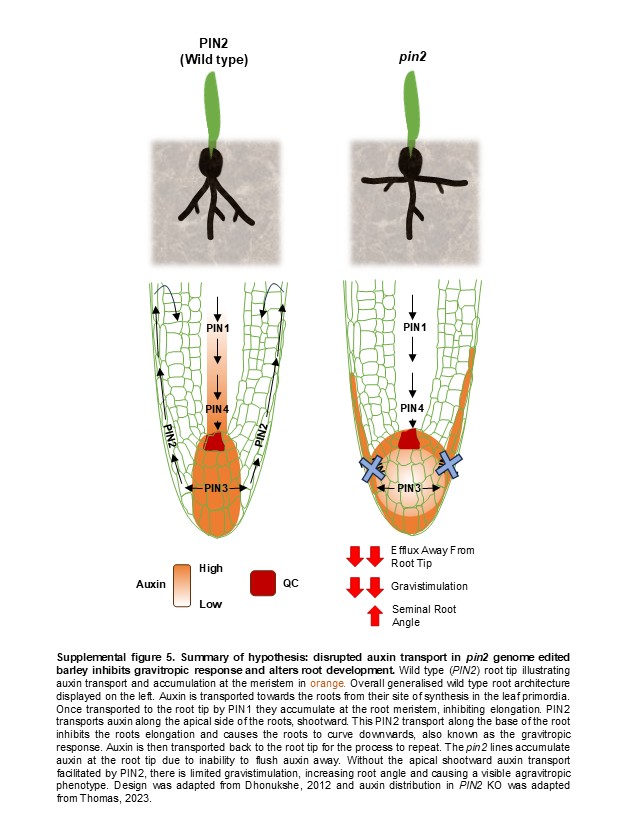

Supplement: Supplementary file 5 — Figure S5. Summary of hypothesis: disrupted auxin transport in pin2 genome edited barley inhibits gravitropic response and alters root development. [file TPG2-18-e70061-s002.jpeg]

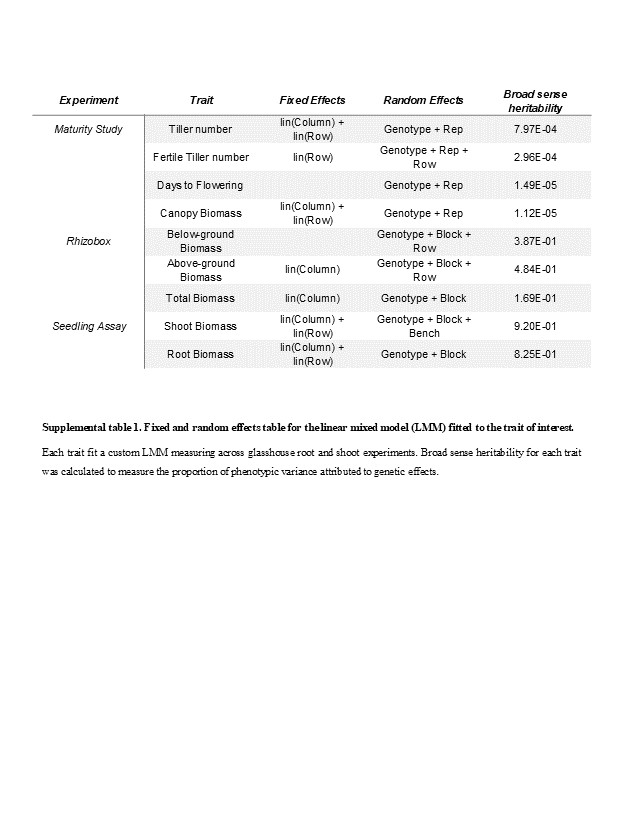

Supplement: Supplementary file 6 — Table S1. Fixed and random effects table for the linear mixed model (LMM) fitted to the trait of interest. [file TPG2-18-e70061-s007.jpeg]

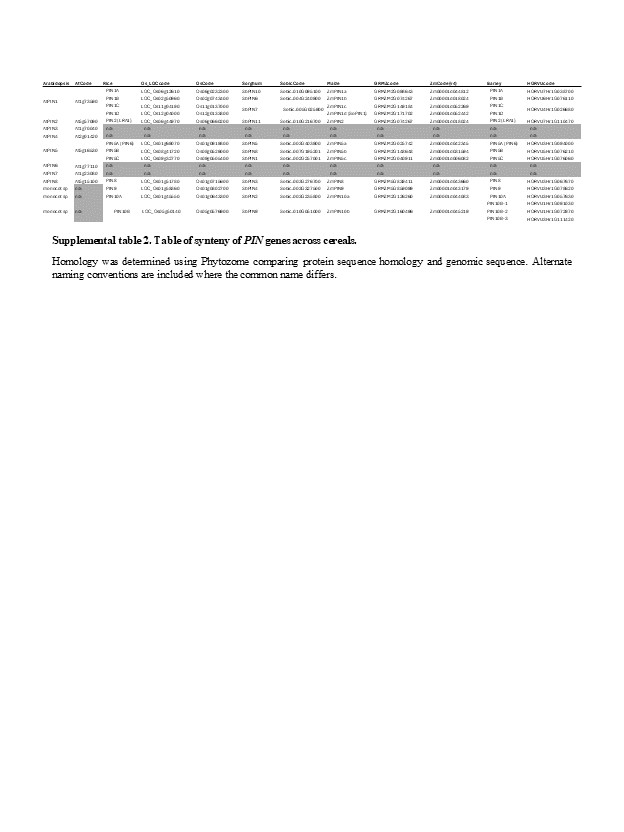

Supplement: Supplementary file 7 — Table S2. Synteny of PIN genes across cereals. [file TPG2-18-e70061-s005.jpeg]

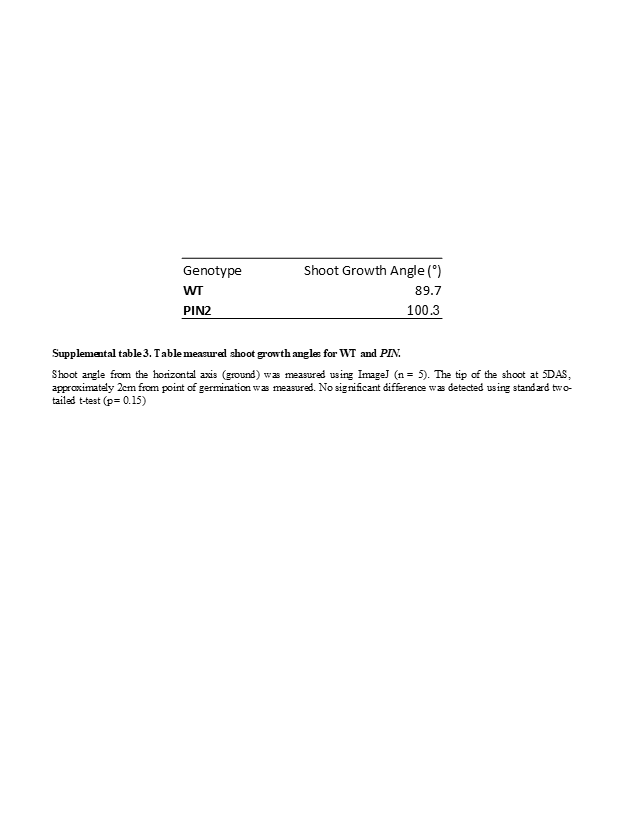

Supplement: Supplementary file 8 — Table S3. Table measured shoot growth angles for WT and PIN2 KO. [file TPG2-18-e70061-s003.jpeg]
